# Epstein-Barr virus-specific T-cell response in pediatric liver transplant recipients: a cross-sectional study by multiparametric flow cytometry

**DOI:** 10.3389/fimmu.2024.1479472

**Published:** 2024-10-24

**Authors:** Ricardo Cuesta-Martín de la Cámara, Andrea Torices-Pajares, Laura Miguel-Berenguel, Keren Reche-Yebra, Esteban Frauca-Remacha, Loreto Hierro-Llanillo, Gema Muñoz-Bartolo, María Dolores Lledín-Barbacho, Almudena Gutiérrez-Arroyo, Ana Martínez-Feito, Eduardo López-Granados, Elena Sánchez-Zapardiel

**Affiliations:** ^1^ Clinical Immunology Department, University Hospital La Paz, Madrid, Spain; ^2^ Lymphocyte Pathophysiology in Immunodeficiencies Group, La Paz Institute for Health Research (IdiPAZ), Madrid, Spain; ^3^ Medicine and surgery Department, Autonomous University of Madrid, Madrid, Spain; ^4^ Paediatric Hepatology Department, University Hospital La Paz, Madrid, Spain; ^5^ European Reference Network (ERN) RARE LIVER, Madrid, Spain; ^6^ European Reference Network (ERN) TransplantChild, Madrid, Spain; ^7^ Microbiology Department, University Hospital La Paz, Madrid, Spain; ^8^ Centre for Biomedical Network Research on rare diseases (CIBERER U767), Madrid, Spain

**Keywords:** liver transplantation, Epstein-Barr virus infections, cellular immunity, flow cytometry, cytokines, surface antigens

## Abstract

**Background:**

Epstein-Barr virus (EBV) specific T-cell response measurement can help adjust immunosuppression in transplant patients with persistent infections. We aim to define T-cell responses against EBV in a cohort of pediatric liver-transplant patients.

**Methods:**

Thirty-eight immunosuppressed pediatric liver-transplant patients (IP) and 25 EBV-seropositive healthy-adult controls (HC) were included in our cross-sectional study. Based on their EBV serological (S) and viral load (VL) status, patients were categorized into IP-S^NEG^, IP-S^POS^VL^NEG^ and IP-S^POS^VL^POS^ groups. T-cell response was assessed at two timepoints by stimulating cells with EBV peptides (PepTivator^®^) and performing intracellular-cytokine and activation-induced marker staining. Background subtraction was used to determine EBV-specific T-lymphocyte frequency.

**Results:**

Polyfunctional CD8+ T cells indicated previous EBV contact (IP-S^NEG^ 0.00% vs IP-S^POS^ 0.04% and HC 0.02%; p=0.001 and p=0.01, respectively). Polyfunctional CD8+CD107a+IFNɣ+IL2-TNFα- profile was increased in serology-positive (IP-S^NEG^ 0.01% vs IP-S_POS_ 0.13% and HC 0.03%; p=0.01 and p=0.50, respectively) and viral-load positive (IP-S^POS^VL^POS^ 0.43% vs IP-S^POS^VL^NEG^ 0.07% and HC 0.03%; p=0.03 and p=0.001, respectively) patients. Central-memory cells were increased among serology-positive adults (IP-S^NEG^ 0.00% vs IP-S^POS^ 0.13% and HC 4.33%; p=0.58 and p=0.002, respectively). At the second timepoint, IP-S^NEG^ patients remained negative (first visit 0.01% vs second visit 0.00%, p=0.44). On the other hand, IP-S^POS^VL^POS^ patients had cleared viral loads and, subsequently, decreased polyfunctional CD8+CD107a+IFNɣ+IL2-TNFα- cells (first visit 0.43% vs second visit 0.10%, p=0.81).

**Conclusion:**

Polyfunctional CD8+ EBV-specific T-cell response allows detecting EBV previous contact in liver-transplant children. %CD8+CD107a+IFNɣ+IL2-TNFα- is increased in patients with positive viral loads. Central memory CD4+ T-cell population more effectively determines prior EBV-exposure in adults.

## 1 Introduction

The progressive improvement of immunosuppressive treatments to prevent graft rejection over the past few decades has contributed to the remarkable improvement in overall graft survival in children receiving liver transplants (1, 2). However, because of this effective immunosuppressive treatment, graft recipients experience a secondary state immunodeficiency, that renders them highly susceptible to infections (3, 4) and malignancies (5).

Most humans are infected with Epstein-Barr virus (EBV), which primarily targets epithelial and B cells, leading to both lytic and latent infections (6). Although EBV has oncogenic potential, it is usually controlled by the immune response. Adults are generally more competent in managing EBV than children, due to a more mature immune system and prior exposure to the virus.

However, in immunosuppressed recipients, EBV reactivation can lead to post-transplant lymphoproliferative disorder (PTLD) (1, 7), a condition characterized by uncontrolled proliferation of EBV-infected cells (8). In liver transplantation, the incidence of PTLD is remarkably higher in pediatric patients (6.3-15.0%) than adults (1.2-2.8%) (9, 10), partially due to their immunologically naïve status for the virus pre-transplantation (seroprevalence in children is approximately 50% vs. 90% in adults) (11) and the incidence of EBV primary infection under immunosuppression (12). In fact, several studies have reported that pre-transplant EBV-seronegative pediatric liver recipients are at higher risk (hazard ratio 12-18) (13) of developing PTLD (14–16).

The Healthcare Working Group of the European Reference Network on Pediatric Transplantation (ERN TransplantChild) has recently published the results of a cross-sectional survey evaluating PTLD strategies for diagnosis and treatment across several pediatric solid organ transplantation programs, from 9 different European countries (17). Over the 2012-2016 period, 1471 pediatric liver transplants were performed and 115 (7.8%) PTLD cases were diagnosed. PTLD preemptive strategies varied across different programs, but all of them included EBV DNA-load measurement by quantitative polymerase chain reaction (qPCR) as the main subrogated biomarker for EBV-specific immunity.

Although EBV-load informative capacity appears to be widely integrated into daily clinical practice, its interpretation for PTLD diagnosis and surveillance is still controversial (18). Actually, no specific EBV viremia cutoff value has been defined to initiate preemptive treatment of PTLD (17). Regarding pediatric liver recipients, the association between high viral load and risk of PTLD development seems to be very poor (19), highlighting the necessity of new biomarkers.

Several techniques have been previously validated in different transplantation settings to estimate T-cell EBV response, being the most standardize one the detection of interferon gamma (IFNɣ) by either enzyme-linked immunospot (ELISpot) (20–23) or enzyme-linked immunosorbent (ELISA)-based (QuantiFERON^®^) assays (24–29). Other promising techniques involve the identification by flow cytometry of antigen-specific cells using mayor histocompatibility complex class I and class II multimers (23, 30–32), intracellular cytokine staining (ICS) (30, 31, 33–42) or activation-induced marker staining (AIMS) (43, 44).

Research on the T-cell compartment against EBV in pediatric liver recipients is scarce and primarily utilizes ELISpot and tetramer assays. There seems to be a correlation between immunosuppression doses and frequency of EBV-specific cells by ELISpot (45–47). Similar results were reported prospectively, measuring cellular response by tetramers (46). Nevertheless, neither tetramers nor dextramers are apparently effective in discriminating transplanted patients according to EBV viral load (32, 48).

Ning et al. used ICS to measure specific T-cell response in two pediatric liver recipients with detectable viral loads and diagnosis of PTLD. Those patients presented a reduction in T-cell polyfunctionality, with an increment in the expression of CD107a and tumor necrosis factor alpha (TNFα) (49). Another study, examining 20 pediatric-transplanted patients (7 liver-graft recipients), presented findings on T-cell response by ICS. Authors reported a significant increment in EBV-specific T cells in PTLD patients during rituximab treatment, which correlated with a reduction in viral load and subsequent control of EBV by T-cell responses following B-cell recovery (50). To our knowledge, the use of AIMS in this field has never been reported, although OX40 (CD134) has been previously defined as a potential biomarker of T-cell activation status in various types of transplants (51, 52).

The aim of this cross-sectional study is to characterize the specific helper and cytotoxic T-cell response to EBV in a cohort of pediatric liver recipients by both ICS and AIMS flow-cytometry methods, and compare it with a cohort of EBV-seropositive healthy adult controls (HC). Furthermore, we aim to identify cellular profiles that allow the discrimination of liver recipients according to both their EBV serological and viral-load status. We hypothesize that pediatric liver recipients controlling EBV will exhibit higher percentages of EBV-specific T cells compared to non-controllers, while also displaying specific cellular profiles.

## 2 Materials and methods

### 2.1 Patients and samples

Our cross-sectional study included 38 immunosuppressed pediatric patients (IP) at University Hospital La Paz, who received a liver graft between March 2018 and November 2022, and 25 EBV seropositive HC. All patients gave informed consent, approved by the ethics committee of our institution (reference PI-4000).

Demographic and clinically relevant information from each patient were collected (**Table 1**). PTLD diagnosis was based on histopathologic criteria. Transplant indication was categorized in five groups (**Table 1**) (53).

**Table 1:** Epidemiologic and clinical features in EBV-seropositive adult healthy controls (HC) and immunosuppressed pediatric liver-transplanted patients, categorized as positive/negative serology status (IP-S^POS^ and IP-S^NEG^, respectively) or positive/negative viral load status (IP-S^POS^VL^POS^ and IP-S^POS^VL^NEG^, respectively).

Characteristics	HC (n=25)	IP-S^POS^ (n=32)	IP-S^NEG^ (n=6)	P-value	HC (n=25)	IP-S^POS^VL^POS^ (n=8)	IP-S^POS^VL^NEG^ (n=24)	P-value
**Sex, n (%)**				0.10				0.07
Male	17 (68)	13 (41)	4 (67)		17 (68)	2 (25)	11 (46)	
Female	8 (32)	19 (59)	2 (33)		8 (32)	6 (75)	13 (54)	
**Age, years (IQR)**	54 (41-62)	5 (3-9)	5 (3-6)	**<0.001**	54 (41-62)	3 (2-4)	5 (4-9)	**<0.001**
**Donor sex, n (%)**				0.62				0.34
Male		8 (25)	3 (50)			1 (13)	7 (29)	
Female		13 (41)	2 (33)			5 (63)	8 (33)	
**Donor age, years (IQR)**		28 (19-34)	27 (15-38)			30 (26-31)	27 (16-34)	
**Type of donor, n (%)**				0.25				0.52
Living donor		10 (31)	4 (67)			3 (38)	7 (29)	
Deceased donor - whole graft		9 (28)	1 (17)			1 (13)	8 (33)	
Deceased donor - split graft		13 (41)	1 (17)			4 (50)	9 (38)	
**ABO compatibility, n (%)**				>0.99				>0.99
Compatible		25 (78)	6 (100)			7 (88)	18 (75)	
Incompatible		3 (9)	0 (0)			1 (13)	2 (25)	
**Indication for transplantation, n (%)**				0.23				0.31
Cholestasis/biliary atresia		19 (59)	3 (50)			6 (75)	13 (54)	
Metabolic diseases		7 (22)	2 (33)			0 (0)	7 (29)	
Cirrhosis (other)		2 (6)	0 (0)			1 (13)	1 (4)	
Severe acute liver failure		2 (6)	0 (0)			1 (13)	1 (4)	
Liver tumours		2 (6)	0 (0)			0 (0)	2 (8)	
Metabolic diseases and liver tumours		0 (0)	1 (17)			0 (0)	0 (0)	
**Time since transplantation, months (IQR)**		40 (31-47)	48 (26-53)	0.47		32 (12-36)	44 (35-47)	**0.002**
**Immunosuppressive treatment, n (%)**				0.47				0.70
CE		5 (16)	0 (0)			2 (25)	3 (13)	
TAC		2 (6)	0 (0)			0 (0)	2 (8)	
CE+TAC		22 (69)	6 (100)			5 (63)	17 (71)	
CE+TAC+MMF		3 (9)	0 (0)			1 (13)	2 (88)	
**TAC blood levels, ng/ml (IQR)**		3.4 (2.7-4.5)	4.4 (3.8-6.7)	0.07		4.1 (3.3-6.5)	3.3 (2.4-4.4)	0.18
**EVB-serology pre-transplantation, n (%)**				**0.03**				0.65
Positive		14 (56)	0 (0)			4 (50)	10 (42)	
Negative		13 (52)	6 (100)			2 (25)	11 (46)	
**CMV-serology pre-transplantation, n (%)**				0.66				>0.99
Positive		15 (47)	2 (33)			4 (50)	11 (46)	
Negative		14 (44)	4 (67)			3 (38)	11 (46)	
**PTLD diagnosis, n (%)**		5 (16)	0 (0)	0.57		2(25)	3(13)	0.58
**Time since diagnosis of PTLD, days (IQR)**		35 (12-133)	NA	NA		8 (6-10)	133 (84-311)	0.20
**Lymphocyte number x10^3^, cells/µL (IQR)**		3.0 (2.4-3.9)	4.0 (2.9-4.3)	0.24		2.7 (2.2-3.3)	3.0 (2.4-4.7)	0.48
**Immune phenotype, % (IQR)**								
CD3+ T lymphocytes		78 (72-85)	72 (65-76)	0.20		74 (69-76)	83 (75-87)	0.11
CD4+ T lymphocytes		45 (38-55)	42 (37-49)	0.55		46 (34-55)	43 (39-56)	>0.99
CD8+ T lymphocytes		22 (17-30)	21 (12-29)	0.41		18 (14-28)	24 (20-36)	0.20
B lymphocytes		8 (6-14)	11 (8-14)	0.55		15 (7-20)	7 (5-9)	0.11
NK lymphocytes		10 (6-16)	19 (10-23)	0.13		13 (8-17)	7 (5-16)	0.33
NKT lymphocytes		0.7 (0.6-1.8)	0.6 (0.5-0.9)	0.20		0.8 (0.5-2.5)	0.7 (0.6-1.8)	0.80

CE, corticosteroids; CMV, Cytomegalovirus; EVB, Epstein-Barr virus; HC, healthy controls; IQR, interquartile range; MMF, mycophenolate mofetil; NA, not applicable; NK, natural killer; NKT, natural killer T; PTLD, post-transplant lymphoproliferative disorder; TAC, tacrolimus. Statistically significant p-values are indicated in bold.

Specific T-cell response against EBV was assessed at two different timepoints, determined by patient availability (median time 3.7 months interquartile range [IQR] 3.2-4.9 between visits). EBV serology and viral load were measured in parallel with each immune response assessment for every patient.

Based on their serological status at the first visit, patients were categorized into EBV seronegative (IP-S^NEG^) and EBV seropositive (IP-S^POS^) individuals; the latter group was further classified according to EBV viral load into negative (IP-S^POS^VL^NEG^) and positive (IP-S^POS^VL^POS^). At the time of the second visit, patients were reclassified based on their updated serological and viral load status at that time. Due to sample exclusions for technical reasons, the immune response was not measured in all samples. ICS was performed to 38/38 (100%) and 28/38 (74%) patients at first and second visit, respectively. AIMS was performed to 27/38 (71%) and 29/38 (76%) patients at first and second visit, respectively, although results for both timepoints were available for only 21 individuals. Among HC participants, ICS and AIMS were successfully performed to 24/25 (96%) and 20/25 (84%) samples, respectively.

Heparinized blood samples from all individuals were collected to isolate peripheral blood mononuclear cells (PBMCs) by density gradient.

### 2.2 Intracellular cytokine staining

Analysis of specific T-cell response to EBV by ICS was assessed as described by Lovelace and Maecker (54). Details of the method are provided in the **Supplementary Material**.

T-cell responses were further categorized as monofunctional, when only one response marker was displayed (CD107a, IFNɣ, Interleukin 2 [IL2] or TNFα), and polyfunctional, when more than one response marker was expressed. Integrated median fluorescence intensity (iMFI) was calculated for each response marker, multiplying MFI by the frequency of the corresponding specific population.

Unstimulated PBMCs background was subtracted from all test samples to obtain the frequency of EBV-specific T lymphocytes. Gating strategy is displayed in **Supplementary Figure S1A**.

### 2.3 Activation-induced cell marker staining

Analysis of AIMS was performed by flow cytometry. Details of the method are provided in the **Supplementary Material**.

CD4+ T lymphocytes were distributed in naïve (Tn, CD27+CD45RO-), effector (Teff, CD27-CD45RO-), central memory (Tcm, CD27+CD45RO+) and effector memory (Tem, CD27-CD45RO+) subpopulations. Memory compartment was calculated by the sum of Tcm and Tem subpopulations.

Unstimulated PBMCs background was subtracted from all test samples to obtain the frequency of EBV-specific T lymphocytes. Gating strategy is displayed in **Supplementary Figure S1B**.

### 2.4 Immunophenotype analysis

Immunophenotype of T, B, natural killer (NK) and natural killer T (NKT) lymphocytes was performed by multiparametric flow cytometry. Details of the method are provided in the **Supplementary Material**.

### 2.5 EBV viral load measurement

EBV viral loads were quantified in whole blood by a specific qPCR assay following manufacturer’s instructions (RealStar^®^ EBV PCR-Kit 1.0, Altona). Results were informed in International Units per milliliter (IU/mL). The negative group for viral load comprised exclusively patients with zero IU/mL.

### 2.6 EBV serology

EBV serological status was determined by a chemiluminescent microparticle immunoassay. Presence of IgM antibodies against viral capsid antigen (VCA) and/or IgG antibodies against VCA and nuclear antigen (EBNA) were measured following manufacturer’s instructions (Abbott, Germany). EBV seropositive status was defined by the positivity of at least one of the analyzed antibodies.

### 2.7 Statistics

Descriptive data are presented as median with IQR. Categorical data are presented as absolute number and proportion (%). The software package Prism 8 (GraphPad, USA) was used for statistical analysis. Significance of differences comparing frequencies was determined by Pearson χ2-test and by *t* test or analysis of variance (Mann Whitney or Kruskal-Wallis tests) when comparing median values. Median frequencies between timepoints were compared by Wilcoxon signed-rank test. Correlation between ICS and AIMS results was assessed by linear regression. P-values under 0.05 were considered significant.

## 3 Results

### 3.1 Demographic and clinical characteristics

A first classification of our cohort (n=38) was performed according to EBV serological status: IP-S^NEG^ (6/38, 16%) and IP-S^POS^ (32/38, 84%). A second distribution of EBV-seropositive patients (n=32) was made according to EBV viral loads: IP-S^POS^VL^POS^ (8/32, 25%) and IP-S^POS^VL^NEG^ (24/32, 75%).

First analysis of our cohort (**Table 1**) showed statistically significant differences when comparing age (HC adults vs. IP) and EBV-serology pre-transplantation (seropositive vs. seronegative). Interestingly, 52% of post-transplant EBV seropositive patients were negative pre-transplantation. When time since transplantation was analyzed, we observed that IP with positive EBV viral loads had been more recently transplanted (IP-S^POS^VL^POS^ 32 months IQR 12-36 vs. IP-S^POS^VL^NEG^ 44 months IQR 35-47, p=0.002). Percentages of T, B and NK subpopulations were similar among groups (**Table 1**).

### 3.2 EBV-specific T-cell response by intracellular cytokine staining

At first visit, EBV-specific %CD3+ T cells by ICS was higher in HC than IP groups (IP-S^NEG^ 0.03% vs. IP-S^POS^ 0.04% and HC 0.06%; p=0.41 and p=0.24, respectively), but differences did not reach statistical signification. Nonetheless, all three groups showed similar positive-control stimulation (IP-S^NEG^ 0.75%, IP-S^POS^ 0.79% and HC 0.45%; p=0.08), indicating that cellular response *in vitro* was not impaired by immunosuppression. Positive-control responses remained comparable when dividing mono/polyfunctional CD4+ and CD8+ T-cell subpopulations (data not shown).

**Figure 1:**
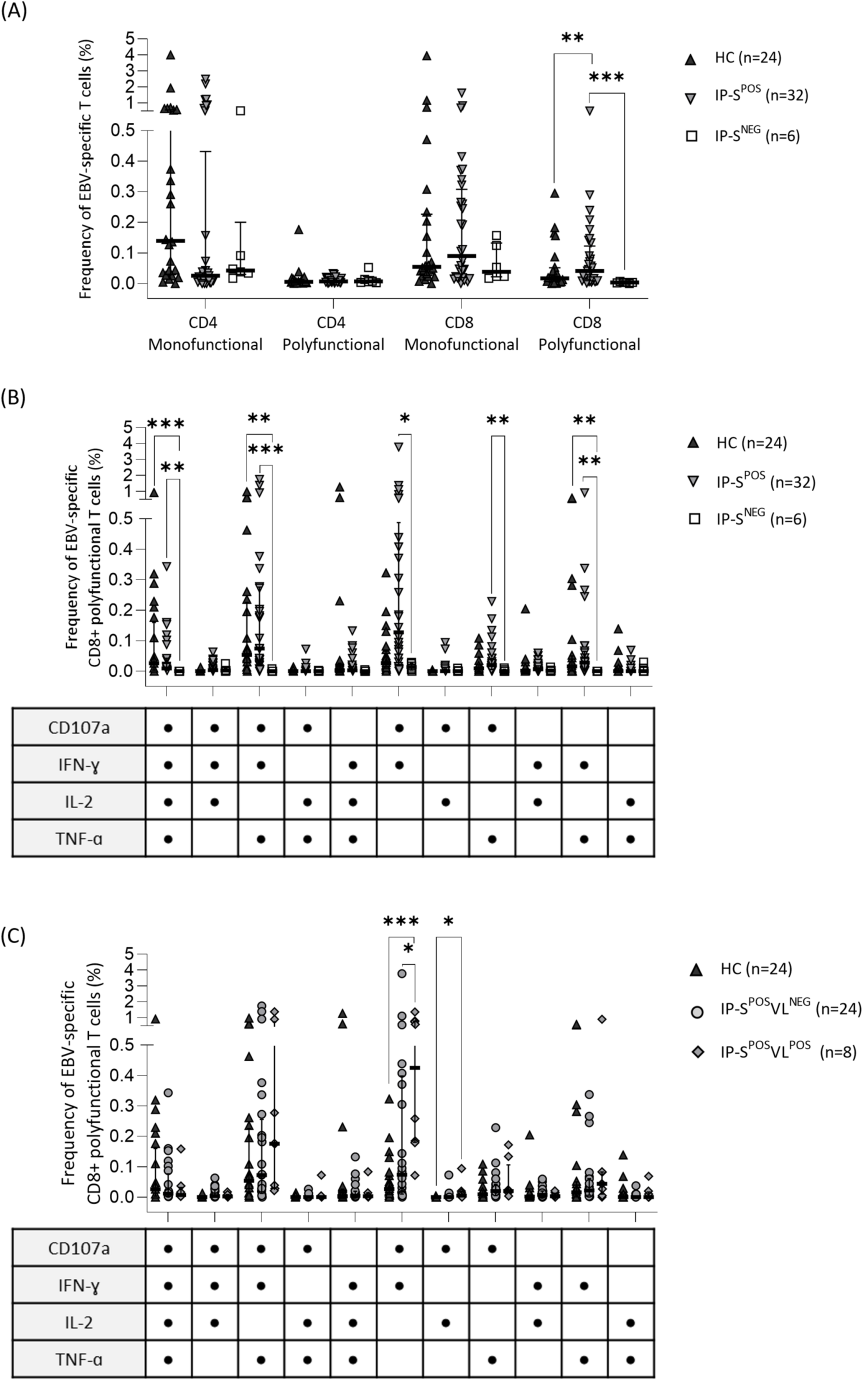
Specific T-cell response to Epstein-Barr virus (EBV) performed by intracellular cytokine staining of CD107a, IFNɣ, IL2 and TNFɑ markers in EBV seropositive healthy adult controls (HC) and immunosuppressed pediatric liver recipients (IP). **(A)** Median frequency of EBV-specific monofunctional (one response marker) or polyfunctional (more than one response markers) CD4+ and CD8+ T cells in HC and IP groups, the last one categorized according to their positive/negative serological status (IP-S^POS^ and IP-S^NEG^, respectively). **(B)** Median frequency of EBV-specific polyfunctional CD8+ T-cell subpopulations according to the different response markers in HC, IP-S^POS^ and IP-S^NEG^ groups. **(C)** Median frequency of EBV-specific polyfunctional CD8+ T-cell subpopulations in HC and IP-S^POS^ groups, the last one segregated according to their positive/negative EBV viral-load status (IP-S^POS^VL^POS^ and IP-S^POS^VL^NEG^, respectively). The same HC group was used as the control group in panels **(A–C)**. Significance levels are denoted as *p-value<0.05, **p-value<0.01 and ***p-value<0.001.

However, when splitting EBV-specific response between T-cell subpopulations (**Figure 1A**), statistically significant differences were observed. Median frequency of monofunctional CD4+ and CD8+ T cells was higher than polyfunctional cells in all groups (**Figure 1A**). Interestingly, although monofunctional responses were detected in higher frequencies, only polyfunctional CD8+ T cells significantly discriminated EBV seronegative patients from seropositive HC and IP groups (IP-S^NEG^ 0.00% vs. IP-S^POS^ 0.04% and HC 0.02%; p=0.01 and p<0.001, respectively; **Figure 1A**).

For a more detailed analysis, the different EBV-specific CD8+ T-cell profiles were analyzed (**Figures 1B, C**). Three different CD8+ polyfunctional profiles differentiated seronegative recipients from both HC and IP seropositive individuals: CD8+CD107a+IFNɣ+IL2+TNFɑ+, CD8+CD107a+IFNɣ+IL2-TNFɑ+ and CD8+CD107a-IFNɣ+IL2-TNFɑ+ (**Figure 1B**). Furthermore, seropositive IP had higher frequencies of EBV-specific CD8+ polyfunctional cells than seronegative IP in two other subsets: CD8+CD107a+IFNɣ+IL2-TNFɑ- and CD8+CD107a+IFNɣ-IL2-TNFɑ+. The profile CD8+CD107a+IFNɣ+IL2-TNFɑ- was the most frequent one (0.13%).

Precisely, this CD8+CD107a+IFNɣ+IL2-TNFɑ- subset significantly discriminated patients with positive viral loads from the rest of individuals (IP-S^POS^VL^POS^ 0.43% vs. IP-S^POS^VL^NEG^ 0.07% and HC 0.03%; p=0.03 and p=0.001, respectively; **Figure 1C**). IP-S^POS^VL^POS^ patients also showed higher %CD8+CD107a+IFNɣ-IL2+TNFɑ- compared to HC, but not to IP-S^POS^VL^NEG^ group (**Figure 1C**). Remarkably, polyfunctional response was more intense than monofunctional response in seropositive individuals (**Supplementary Table S1**). All three cytokine markers IFNɣ, IL2 and TNFɑ had significantly higher iMFI values in polyfunctional response, both in CD4+ and CD8+ T cells, whereas CD107a only showed higher intensity in polyfunctional CD8+ T cells (**Supplementary Table S1**). In line with our previous results, CD8+ polyfunctional subpopulation allowed discriminating seropositive from seronegative status, according to iMFI from all four response markers (**Table 2**). However, regarding EBV viral load, only total (mono and polyfunctional) CD107a iMFI on CD8+ T cells significantly differentiated IP with positive viral loads from the other groups (IP-S^POS^VL^POS^ 123,398 vs. IP-S^POS^VL^NEG^ 20,708 and HC 21,207; p=0.03 and p=0.01, respectively).

**Table 2:** Median of the integrated median fluorescence intensity (iMFI) for each marker (CD107a, IFNɣ, IL2 or TNFα) in both polyfunctional CD4+ and CD8+ T cells in EBV-seropositive adult healthy controls (HC) and immunosuppressed pediatric liver-transplanted patients, categorized as positive/negative serology status (IP-S^POS^ and IP-S^NEG^, respectively) or positive/negative viral load status (IP-S^POS^VL^POS^ and IP-S^POS^VL^NEG^, respectively).

iMFI		HC (n=24)	IP-S^POS^ (n=32)	IP-S^NEG^ (n=6)	P-value	HC (n=24)	IP-S^POS^VL^POS^ (n=8)	IP-S^POS^VL^NEG^ (n=24)	P-value
T-cell subset	Parameter								
**Polyfunctional CD4+ T cells**	CD107a+	1,060	1,813	2,588	0.43	1,060	2,651	1,813	0.35
	IFNɣ+	475	179	49	0.07	475	181	179	0.13
	IL2+	122	93	237	0.69	122	95	93	0.93
	TNFα+	1,248	1,422	1,838	0.84	1,248	1,705	1422	0.85
**Polyfunctional CD8+ T cells**	CD107a+	17,538	42,771	373	**0.002**	17,538	105,001	18,628	**0.03**
	IFNɣ+	3,366	7,714	36	**0.002**	3,366	10,042	5,228	0.34
	IL2+	377	424	0	**0.001**	377	329	488	1.00
	TNFα+	7,370	8,307	0	**0.001**	7,370	11,509	7,391	0.86

Statistically significant p-values are indicated in bold.

To simplify cytometry panels, we studied whether CD8+ T cells expressing only CD107a and IFNɣ markers could be distinctive, regardless of other cytokines. As expected, %CD8+CD107a+IFNɣ+ T cells were significantly higher among seropositive individuals compared to seronegative IP (IP-S^NEG^ 0.02% vs. IP-S^POS^ 0.37% and HC 0.12%; p=0.002 and p=0.04, respectively). However, considering EBV viral load, although the frequency of specific cells was also increased within positive IP group, differences were significant compared to controls, but not to negative patients (IP-S^POS^VL^POS^ 0.70% vs. IP-S^POS^VL^NEG^ 0.19% and HC 0.12%; p=0.10 and p=0.03, respectively).

Finally, we explored whether CD3+IFNɣ+ T cells, the main population targeted by other methods, allowed discrimination of serology and/or viral-load status. While frequency of CD3+IFNɣ+ T cells was insufficient for serology discrimination (IP-S^NEG^ 0.10% vs. IP-S^POS^ 0.31% and HC 0.12%; p=0.31 and p>0.99, respectively), iMFI was significantly higher in seropositive than seronegative individuals (IP-S^NEG^ 182 vs. IP-S^POS^ 3,628 and HC 1,380; p=0.003 and p=0.01, respectively). When comparing viral-load status no statistically differences were reached either with CD3+IFNɣ+ frequency (IP-S^POS^VL^POS^ 0.37% vs. IP-S^POS^VL^NEG^ 0.24% and HC 0.12%; p>0.99 and p=0.40, respectively) or iMFI (IP-S^POS^VL^POS^ 3,834 vs. IP-S^POS^VL^NEG^ 2,809 and HC 1,380; p=0.62 and p=0.82, respectively).

### 3.3 EBV-specific T cell response by activation-induced cell marker staining

**Figure 2:**
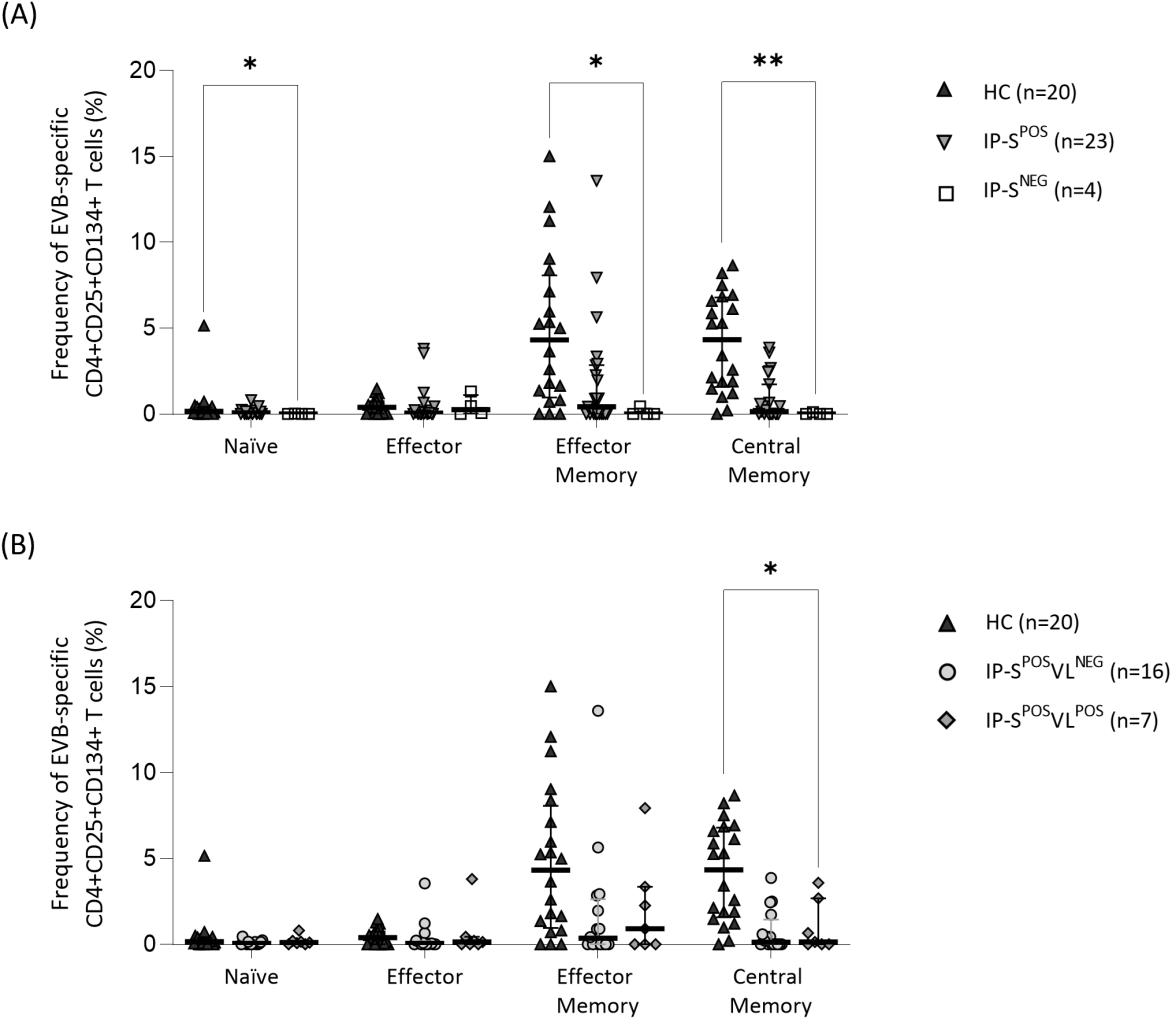
Specific T-cell response to Epstein-Barr virus (EBV) performed by activation-induced marker staining (CD4+CD25+CD134+) in EBV seropositive healthy adult controls (HC) and immunosuppressed pediatric liver recipients (IP). **(A)** Median frequency of EBV-specific CD4+CD25+CD134+ T cells in HC and IP groups, the last one categorized according to their positive/negative serological status (IP-S^POS^ and IP-S^NEG^, respectively). **(B)** Median frequency of EBV-specific CD4+CD25+CD134+ cells in HC and IP-S^POS^ groups, the last one segregated according to positive/negative EBV viral-load status (IP-S^POS^VL^POS^ and IP-S^POS^VL^NEG^, respectively). The same HC group was used as the control group in both panels **(A, B)**. Significance levels are denoted as *p-value<0.05, **p-value<0.01 and ***p-value<0.001.

At first visit, differences were found in EBV-specific T-cell frequencies by AIMS between HC and IP groups (**Figures 2A, B**). As expected, CD4+ Tn EBV-specific subset was the lowest within each group, compared to Teff and memory compartment (**Figures 2A, B**). On the other hand, seropositive groups (**Figure 2A**) had higher frequency of EBV-specific memory compartment than Teff cells (IP-S^POS^ memory compartment 0.99% vs. Teff 0.04%, p=0.12; HC memory compartment 8.24% vs. Teff 0.37%, p<0.001), although differences were statistically significant only within HC group. Interestingly, seronegative IP showed higher frequencies of Teff than memory EBV-specific cells (memory compartment 0.00% vs. Teff 0.25%, p=0.14), although differences did not reach statistical signification.

Compared to IP seronegative group, percentages of EBV-specific cells were higher in HC group for Tn (0.14% vs. IP-S^NEG^ 0.00%, p=0.03), Tem (4.31% vs. IP-S^NEG^ 0.00%, p=0.01) and Tcm (4.33% vs. IP-S^NEG^ 0.00%, p=0.002) subpopulations (**Figure 2A**). However, comparing frequencies between seropositive and seronegative IP did not yield any statistically significant differences. Again, positive-control stimulation in CD4+ T cells was comparable by AIMS (IP-S^NEG^ 24.52%, IP-S^POS^ 32.67% and HC 26.42%; p=0.62).

We next studied seropositive IP grouped by viral-load status (**Figure 2B**) and observed that %CD4+ Tcm cells was significantly higher in HC than seropositive IP with detectable EBV (IP-S^POS^VL^POS^ Tcm 0.13% vs. HC Tcm 4.32%, p=0.02). Interestingly, median %CD4+ Tem cells in seropositive IP-S^POS^VL^POS^ was higher than in IP-S^POS^VL^NEG^ (0.90% vs. 0.34%, respectively; p>0.99), although no significant differences were found.

**Figure 3:**
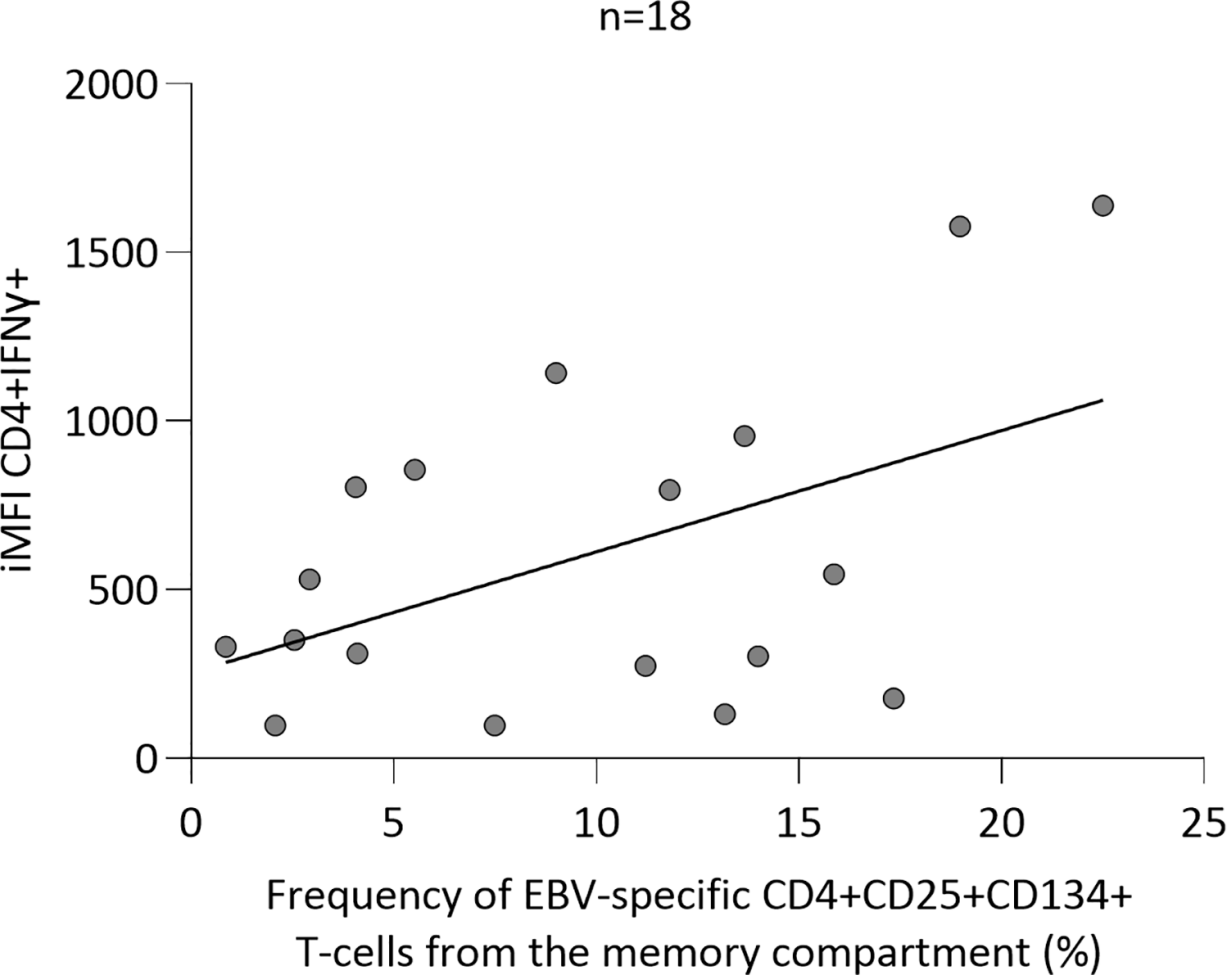
Linear regression analysis between integrated median fluorescence intensity (iMFI) of Epstein-Barr virus (EBV)-specific CD4+IFNɣ+ T cells measured by intracellular cytokine staining and EBV-specific CD4+CD25+CD134+ central memory T (Tcm) cells measured by activation-induced marker staining.

Finally, we further investigated the potential correlation between the parameters defined by ICS (%CD8+CD107a+IFNɣ+IL2-TNFɑ-) and AIMS (%CD4+CD134+CD25+ Tcm), which effectively distinguished positive from negative individuals. We noted that those parameters exhibited no correlation (data not shown), likely due to the comparison involving distinct T-cell subpopulations. Thus, a potential correlation between EBV-specific CD4+ T cells by ICS (% and iMFI CD4+INFɣ) and AIMS (%CD4+CD134+CD25+ Tcm) techniques was next sought, although we did not observe any correlation, by either frequency or iMFI (data not shown). However, within HC group, after excluding the data from one individual lacking EBV-specific CD4+CD134+CD25+ memory T cells, a significant correlation between iMFI CD4+INFɣ by ICS and %CD4+CD134+CD25+ by AIMS (r^2^ = 0.24 and p=0.04, **Figure 3**) was found.

### 3.4 EBV-specific T cell response at two different timepoints

EBV-specific response was measured on a second visit by both techniques. Serological and viral-load status was re-evaluated and patients were reclassified accordingly. Tacrolimus blood levels remained similar at first and second timepoints in all three groups (data not shown).

At the second timepoint, identical results to those reported at the first visit were found when comparing frequencies of EBV-specific cells detected by ICS and AIMS. We confirmed that only polyfunctional CD8+ specific T cells significantly discriminated EBV seronegative patients from seropositive individuals (IP-S^NEG^ 0.00% vs. IP-S^POS^ 0.03% and HC 0.02%; p=0.01 and p=0.02, respectively). Likewise, %EBV-specific cells from HC were higher than those detected in seronegative patients for Tn (0.14% vs. 0.01%, p=0.05), Tem (4.31% vs. 0.00%, p=0.01) and Tcm (4.33% vs. 0.00%, p=0.003) subpopulations.

**Figure 4:**
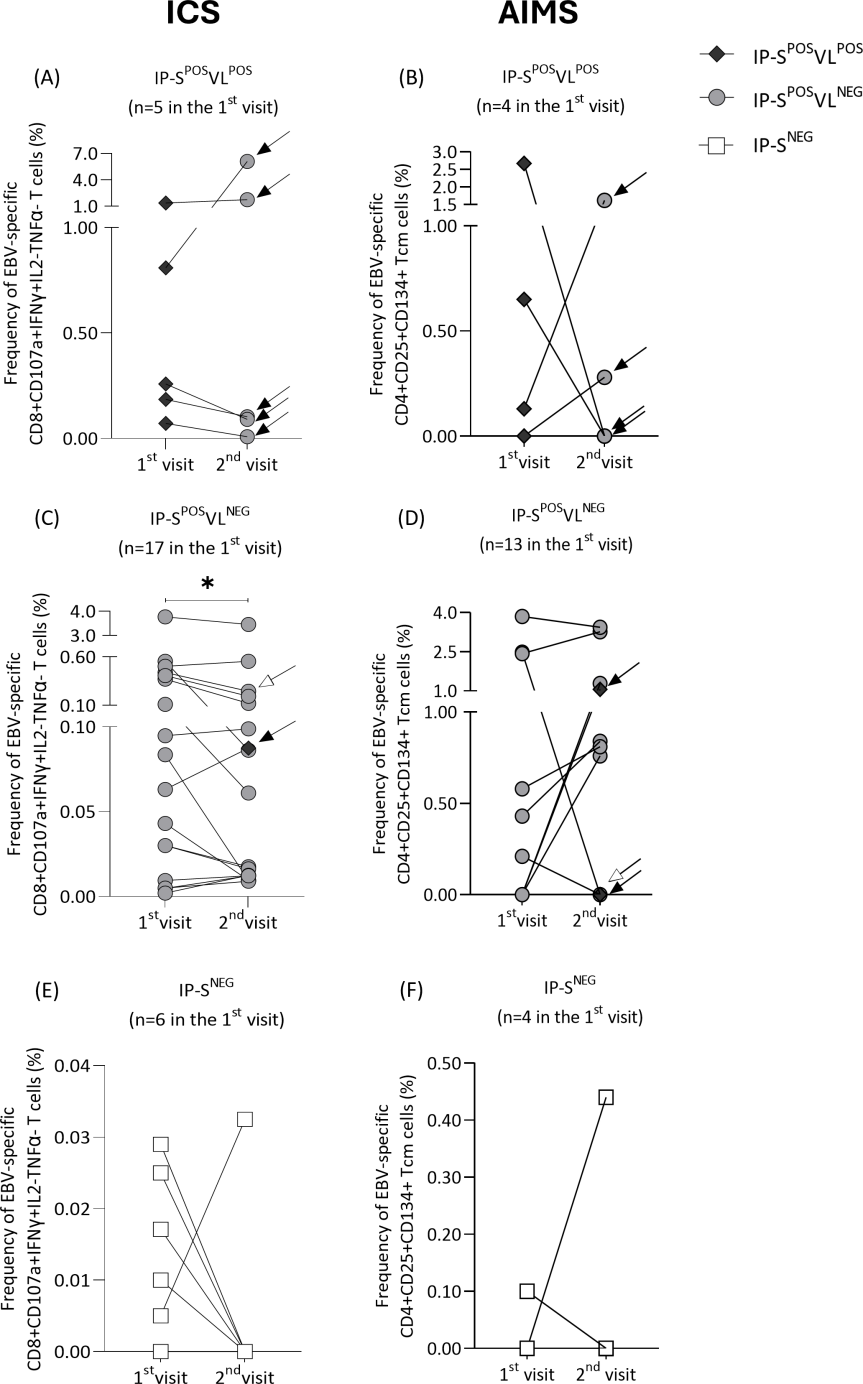
Frequency of Epstein-Barr virus (EBV)-specific CD8+CD107a+IFNɣ+IL2-TNFɑ- T cells **(A, C, E)** and CD4+CD25+CD134+ central memory T (Tcm) cells **(B, D, F)** measured at two different timepoints (1^st^ and 2^nd^ visit) in immunosuppressed pediatric liver recipients (IP). Patients were classified at their first visit according to their positive/negative EBV serological and viral-load status: IP-S^POS^VL^POS^ **(A, B)**, IP-S^POS^VL^NEG^ **(C, D)** and IP-S^NEG^ **(E, F)** groups. At the second visit, their updated serological and viral-load status is represented by rhomboid, circular or square markers, respectively. Changes in their classification at second visit are marked with a black arrow. Patients that suffered changes in his immunosuppression regimen at second visit are marked with a white arrow. Significance levels are denoted as *p-value<0.05, **p-value<0.01 and ***p-value<0.001.

Regarding IP-S^POS^VL^POS^ group, all recipients with detectable EBV at first timepoint cleared viral loads at the second visit (median time 3.5 months IQR 2.2-5.3 between timepoints). Consequently, median %CD8+CD107a+IFNɣ+IL2-TNFɑ- (first visit 0.26% vs. second visit 0.10%, p=0.81; **Figure 4A**) and %CD4+CD25+CD134+ Tcm cells (first visit 0.39% vs. second visit 0.14%, p=0.88; **Figure 4B**) decreased at the second timepoint, although no significant differences were found.

On the other hand, only two IP-S^POS^VL^NEG^ patients at first timepoint had detectable EBV at the second visit (black arrows in **Figures 4C, D**) (median time 3.7 months IQR 3.3-4.8 between timepoints). Interestingly, significant differences were found by ICS (first visit 0.08% vs. second visit 0.06%, p=0.04; **Figure 4C**), but not by AIMS (first visit 0.00% vs. second visit 0.76%, p=0.31; **Figure 4D**). Frequency of CD8+CD107a+IFNɣ+IL2-TNFɑ- cells decreased or remained similar for all patients, except for the individual who tested positive for EBV at the second visit, whose frequency increased from 0.06% to 0.09% (black arrow in **Figure 4C**). This patient’s frequency of specific CD4+CD25+CD134+ Tcm cells also increased from 0.00% to 1.06% (black arrow in **Figure 4D**). Nevertheless, that variation in the specific response by AIMS was not observed in the other patient who tested positive for viral load at the second timepoint (black arrow in **Figure 4D**). One IP-S^POS^VL^NEG^ patient had his immunosuppression regimen changed between visits, incorporating mycophenolate to his treatment with corticosteroids and tacrolimus. Interestingly, frequency of EBV-specific T cells by ICS decreased from 0.44% to 0.25%, while frequency of CD4+CD25+CD134+ Tcm cells remained at 0.00% (white arrows in **Figures 4C, D**).

Finally, all seronegative patients (**Figures 4E, F**) kept their negative serological status at the second visit (median time of 3.9 months IQR 3.6-4.0 between timepoints). Consequently, no differences in EBV-specific response were found, either by ICS (0.01% vs. 0.00%, p=0.44; **Figure 4E**) or AIMS (0.00% vs. 0.00%, p>0.99; **Figure 4F**).

## 4 Discussion

In our study, we have first explored EBV-specific T-cell response combining ICS and AIMS techniques in a cohort of pediatric liver transplanted recipients. We found significant differences in polyfunctional CD8+ T-cell response between EBV-seronegative and seropositive individuals, and among patients with positive and negative viral loads.

Firstly, we found higher percentages of monofunctional than polyfunctional EBV-specific T cells. This could be attributed to cross-reactivity resulting from heterologous immunity (55), although recent studies state that it is less generalized than previously reported (56). Since we have also confirmed that monofunctional responses showed lower iMFI (49), we can attribute this result to a potential unspecific bystander activation *in vitro*. The presence of monofunctional EBV-specific cells among seronegative patients provides additional support for that hypothesis.

The predominance of CD8+ over CD4+ T-cell responses in controlling EBV infection is well documented (49, 57). Although both CD4+ and CD8+ T cells show polyfunctional responses after primary infection, only the CD8+ polyfunctional subset increases over time (58). Accordingly, we observe that polyfunctional CD8+ T cells are significantly increased in seropositive individuals (**Figure 1B**). Remarkably, we expected a reduction in the frequency of this population in patients with detectable viral loads, in concordance with Ning et al., who demonstrated this in two pediatric liver recipients with PTLD (49). Conversely, IP-S^POS^VL^POS^ in our cohort showed high %CD8+ EBV-specific cells (**Figure 1C**), including 2 patients who were studied at the time of PTLD diagnosis.

The increment of CD8+CD107a+IFNɣ+IL2-TNFɑ- in recipients with detectable EBV viral loads reflects the critical role of cytotoxicity, as indicated by CD107a, and the antiviral function of IFNɣ in the response against EBV. Frequent EBV reactivation might inflate this cell compartment, exhausting and rendering cells dysfunctional, thus requiring larger numbers to control the virus (59). Previously suggested explanation for pediatric graft recipients carrying chronically high EBV loads involves an exhausted phenotype (23). We did not include exhaustion markers in our study, but we consider that they would be useful to better describe viral responses in future investigations. Other protocols, such as expanding T cells in the presence of EBV peptides for 7-10 days before analysis (60), could also be applied. However, we chose shorter incubation times (54), as this approach was more compatible with the workflow of our routine laboratory.

We further evaluated CD107a combined with IFNɣ, as exclusive response markers to distinguish patients with positive viral loads. While %CD8+CD107a+IFNɣ+ helped in serostatus discrimination, it did not show significant differences in EBV viral loads, likely due to our limited sample size. Similar results were reported by Wilsdorf et al., who measured intracellular IFNɣ after EBV-peptide stimulus in pediatric transplanted patients with PTLD (4/16 liver-graft recipients) or positive viral-loads (3/4 liver-graft recipients) and 18 HC. Median %CD4+IFNɣ+ and %CD8+IFNɣ+ was higher in recipients with EBV reactivation, yet they did not find significant differences either (50).

In our cohort, %CD3+IFNɣ+ cells do not differentiate seropositive from seronegative patients, contrary to prior studies using ELISpot (47). Instead, we found that the intensity of IFNɣ response in CD3+ T cells effectively differentiated these two groups. While no equivalent parameter to iMFI has been described in ELISpot assays, mean spot size could be comparable (61). Our findings may be influenced by the constraints of our sample size, but conducting additional studies to explore iMFI further would be valuable.

On the other hand, the use of AIMS to measure specific viral response is not extended, although it has been validated in HC for Varicella-Zoster Virus, Cytomegalovirus, EBV (44) and Hepatitis C (43). Regarding EBV, it seems feasible to distinguish seronegative from seropositive individuals measuring %CD4+CD134+CD25+ specific-cells (44). We confirmed these findings by examining Tcm and Tem CD4+ subsets, consistent with the predominant memory CD4+ response to EBV (62). Interestingly, we detected specific CD4+ Teff cells in seronegative individuals, probably reflecting antigen exposure in some patients (63), although we cannot exclude unspecific activation, since percentages were similar among groups. The increase of EBV-specific CD4+ Tn in HC (**Figure 1A**) has been previously reported as a genuine memory population transitioning to express naïve surface markers (62). This stem memory T-cell population (CCR7+CD27+CD45RO-) shares some features with Tn and requires staining with specific markers (CD95) for proper selection (64). These cells emerge rapidly post-antigen exposure, transitioning into effector cells, while retaining self-renewal and multipotent abilities, making them ideal for adoptive T-cell therapies, including EBV infection in transplant recipients (65, 66).

Due to the age gap, memory response in HC is the highest, reflecting repeated exposures to EBV antigens over their lifetimes, which expands the clonal repertoire against the virus (23). Interestingly, Tem specific subset is increased in patients with active viral replication, consistent with findings by Amyes et al. (67). They observed a primary burst of CD4+ Teff cells in response to EBV, persisting throughout the chronic phase of infection. However, our stimulation with a cocktail of lytic and latent EBV peptides does not differentiate between viral phases (58, 67).

Regarding correlation between ICS and AIMS, Sadler et al. demonstrated that EBV-specific production of IFNɣ significantly correlated with %CD4+CD134+CD25+ cells in HC (44). While we did not replicate this result, we found %CD4+CD134+CD25+ cells from memory compartment correlated with CD4+ response measured by IFNɣ iMFI, confirming AIMS reliability to infer specific CD4+ T-cell response. Further studies on CD8+ T-cell activation markers, such as CD38 and HLA-DR (44), are recommended.

At the second visit, we confirmed that polyfunctional CD8+ EBV-specific T-cell response detects serology and viral-load positive individuals. Furthermore, we replicated our findings on CD4+ Tcm population in adults. Compared to first visit, we observed changes in EBV-specific cellular response among patients with viral-load status shifts, significant in the largest sample group (**Figure 4C**). Interestingly, tacrolimus blood levels remained similar between visits, questioning the effect of immunosuppression treatment. Positive control stimulus elicited a similar reaction in HC and immunosuppressed patients, suggesting that anti-CD3/28 beads override immunosuppression. Similarly, Arasaratnam et al. found comparable IFNɣ production by Staphylococcal enterotoxin B in pediatric liver recipients post-immunosuppression (45).

On the other hand, other authors observed that immunosuppression treatment modifications for PTLD management lead to changes in frequency of EBV-specific cells detected by ELISpot (45–47). Moreover, OX40 has been postulated as an indicator of the immunosuppressive status of patients after stem cell transplantation (68), although Lamb et al. reported a recipient of stem cell transplantation whose changes in the immunosuppressive treatment did not reflect differences in %CD4+CD134+ cells (69). To elucidate the potential use of ICS or AIMS in evaluating immunosuppressive status of transplanted patients further prospective studies are needed.

In conclusion, our study reveals significant insights into EBV-specific T-cell responses in pediatric liver transplant recipients. We demonstrated that polyfunctional CD8+ T cells were markedly increased in seropositive individuals, underscoring their role in controlling EBV infection. Despite a limited sample size, our findings support the utility of CD107a/IFNɣ response markers for distinguishing EBV serostatus and viral load. Additionally, OX40 proved reliable in assessing CD4+ memory responses, suggesting its potential for broader application in evaluating antiviral immunity. Further prospective research is recommended to refine our understanding of EBV-specific T-cell dynamics in transplant recipients.

## Data Availability

The original contributions presented in the study are included in the article/**Supplementary Material**. Further inquiries can be directed to the corresponding author.

## Supplementary material

Supplementary Figure 1 Flow-cytometry gating strategy for **(A)** intracellular cytokine staining or **(B)** activation-induced marker staining in a representative healthy control. Cells were left unstimulated (NC) or stimulated using a positive control (PC) or Epstein-Barr virus peptides (EBV).

## References

[1] Jain A Mazariegos G Kashyap R Kosmach-Park B Starzl TE Fung J. Pediatric liver transplantation. A single center experience spanning 20 years. Transplant. (2002) 73:941–7. doi: 10.1097/00007890-200203270-00020 PMC2975975 11923697

[2] Jara P Hierro L. Trasplante hepático infantil. Resultados a largo plazo. Gastroenterol Hepatol. (2010) 33:398–410. doi: 10.1016/j.gastrohep.2009.11.004 20122757

[3] Fishman JA. Infection in organ transplantation. Am J Transplant. (2017) 17:856–79. doi: 10.1111/ajt.14208 28117944

[4] Roberts MB Fishman JA. Immunosuppressive agents and infectious risk in transplantation: managing the “Net state of immunosuppression. Clin Infect Dis. (2021) 73:e1302–17. doi: 10.1093/cid/ciaa1189 PMC8561260 32803228

[5] Domhan S Zeier M Abdollahi A. Immunosuppressive therapy and post-transplant Malignancy. Nephrol Dial Transplant. (2008) 24:1097–103. doi: 10.1093/ndt/gfn605 18978068

[6] Huang W Bai L Tang H. Epstein-Barr virus infection: the micro and macro worlds. Virol J. (2023) 20:220. doi: 10.1186/s12985-023-02187-9 37784180 PMC10546641

[7] Green M Webber S. Posttransplantation lymphoproliferative disorders. Pediatr Clin North Am. (2003) 50:1471–91. doi: 10.1016/S0031-3955(03)00127-5 14710788

[8] Martinez OM Krams SM. The immune response to epstein barr virus and implications for posttransplant lymphoproliferative disorder. Transplant. (2017) 101:2009–16. doi: 10.1097/TP.0000000000001767 PMC5568952 28376031

[9] Eshraghian A Imanieh MH Dehghani SM Nikeghbalian S Shamsaeefar A Barshans F. Post-transplant lymphoproliferative disorder after liver transplantation: Incidence, long-term survival and impact of serum tacrolimus level. WJG. (2017) 23:1224. doi: 10.3748/wjg.v23.i7.1224 28275302 PMC5323447

[10] Taylor AL Marcus R Bradley JA. Post-transplant lymphoproliferative disorders (PTLD) after solid organ transplantation. Crit Rev Oncol/Hematol. (2005) 56:155–67. doi: 10.1016/j.critrevonc.2005.03.015 15979320

[11] Trottier H Buteau C Robitaille N Duval M Tucci M Lacroix J. Transfusion-related Epstein-Barr virus infection among stem cell transplant recipients: a retrospective cohort study in children. Transfusion. (2012) 52:2653–63. doi: 10.1111/j.1537-2995.2012.03611.x 22420319

[12] Green M Michaels MG. Epstein–barr virus infection and posttransplant lymphoproliferative disorder. Am J Transplant. (2013) 13:41–54. doi: 10.1111/ajt.12004 23347213

[13] San-Juan R Comoli P Caillard S Moulin B Hirsch HH Meylan P. Epstein-Barr virus-related post-transplant lymphoproliferative disorder in solid organ transplant recipients. Clin Microbiol Infect. (2014) 20:109–18. doi: 10.1111/1469-0691.12534 24475976

[14] Barış Z Özçay F Yılmaz Özbek Ö Haberal N Sarıalioğlu F Haberal M. A single-center experience of post-transplant lymphoproliferative disorder (PTLD) cases after pediatric liver transplantation: Incidence, outcomes, and association with food allergy. Turk J Gastroenterol. (2018) 29:354–60. doi: 10.5152/tjg.2018.17731 PMC6284654 29755021

[15] Quintero Bernabeu J Juamperez J Mercadal-Hally M Larrarte King M Gallego Melcon S Gros Subias L. Epstein–Barr virus-associated risk factors for post-transplant lymphoproliferative disease in pediatric liver transplant recipients. Pediatr Transplant. (2022) 26(6):e14292. doi: 10.1111/petr.14292 35466492

[16] Wu JF Ho MC Ni YH Chen HL Lu CY Hsu HY. Timing of epstein-barr virus acquisition and the course of posttransplantation lymphoproliferative disorder in children. Transplant. (2009) 87:758–62. doi: 10.1097/TP.0b013e318198d645 19295323

[17] Baker A Frauca Remacha E Torres Canizales J Bravo-Gallego LY Fitzpatrick E Alonso Melgar A. Current practices on diagnosis, prevention and treatment of post-transplant lymphoproliferative disorder in pediatric patients after solid organ transplantation: results of ERN transplant Child healthcare working group survey. Children. (2021) 8:661. doi: 10.3390/children8080661 34438552 PMC8394841

[18] Allen UD Preiksaitis JK. The AST Infectious Diseases Community of Practice. Post-transplant lymphoproliferative disorders, Epstein-Barr virus infection, and disease in solid organ transplantation: Guidelines from the American Society of Transplantation Infectious Diseases Community of Practice. Clin Transplant. (2019) 33(9):e13652. doi: 10.1111/ctr.13652 31230381

[19] Green M Soltys K Rowe DT Webber SA Mazareigos G. Chronic high Epstein-Barr viral load carriage in pediatric liver transplant recipients. Pediatr Transplant. (2009) 13:319–23. doi: 10.1111/j.1399-3046.2008.00926.x 18397216

[20] Abate D Saldan A Fiscon M Cofano S Paciolla A Furian L. Evaluation of Cytomegalovirus (CMV)–Specific T Cell Immune Reconstitution Revealed That Baseline Antiviral Immunity, Prophylaxis, or Preemptive Therapy but not Antithymocyte Globulin Treatment Contribute to CMV-Specific T Cell Reconstitution in Kidney Transplant Recipients. J Infect Dis. (2010) 202:585–94. doi: 10.1086/654931 20594105

[21] Calarota SA Chiesa A Zelini P Comolli G Minoli L Baldanti F. Detection of Epstein-Barr virus-specific memory CD4 ^+^ T cells using a peptide-based cultured enzyme-linked immunospot assay. Immunology. (2013) 139:533–44. doi: 10.1111/imm.2013.139.issue-4 PMC3719070 23560877

[22] Jarque M Crespo E Melilli E Gutiérrez A Moreso F Guirado L. Cellular immunity to predict the risk of cytomegalovirus infection in kidney transplantation: A prospective, interventional, multicenter clinical trial. Clin Infect Dis. (2020) 71(9):2375–85. doi: 10.1093/cid/ciz1209 32076718

[23] Macedo C Webber SA Donnenberg AD Popescu I Hua Y Green M. EBV-specific CD8+ T cells from asymptomatic pediatric thoracic transplant patients carrying chronic high EBV loads display contrasting features: activated phenotype and exhausted function. J Immunol. (2011) 186:5854–62. doi: 10.4049/jimmunol.1001024 PMC4165085 21460204

[24] Kumar D Chernenko S Moussa G Cobas I Manuel O Preiksaitis J. Cell-mediated immunity to predict cytomegalovirus disease in high-risk solid organ transplant recipients. Am J Transplant. (2009) 9:1214–22. doi: 10.1111/j.1600-6143.2009.02618.x 19422346

[25] Kumar D Mian M Singer L Humar A. An interventional study using cell-mediated immunity to personalize therapy for cytomegalovirus infection after transplantation. Am J Transplant. (2017) 17:2468–73. doi: 10.1111/ajt.14347 28500691

[26] Lisboa LF Kumar D Wilson LE Humar A. Clinical utility of cytomegalovirus cell-mediated immunity in transplant recipients with cytomegalovirus viremia. Transplant. (2012) 93:195–200. doi: 10.1097/TP.0b013e31823c1cd4 22179399

[27] Manuel O Husain S Kumar D Zayas C Mawhorter S Levi ME. Assessment of cytomegalovirus-specific cell-mediated immunity for the prediction of cytomegalovirus disease in high-risk solid-organ transplant recipients: A multicenter cohort study. Clin Infect Dis. (2013) 56:817–24. doi: 10.1093/cid/cis993 23196955

[28] Poglajen G Zemljic G Frljak S Okrajšek R Šebeštjen M Cerar A. Quantiferon-CMV guided virostatic prophylaxis after heart transplantation. J Heart Lung Transplant. (2019) 38:S119. doi: 10.1016/j.healun.2019.01.279 32005602

[29] Westall GP Cristiano Y Levvey BJ Whitford H Paraskeva MA Paul E. A randomized study of quantiferon CMV-directed versus fixed-duration valganciclovir prophylaxis to reduce late CMV after lung transplantation. Transplant. (2019) 103:1005–13. doi: 10.1097/TP.0000000000002454 30247316

[30] Benz C Utermöhlen O Wulf A Villmow B Dries V Goeser T. Activated virus-specific T cells are early indicators of anti-CMV immune reactions in liver transplant patients. Gastroenterology. (2002) 122:1201–15. doi: 10.1053/gast.2002.33021 11984506

[31] Sund F Lidehäll AK Claesson K Foss A Tötterman TH Korsgren O. CMV-specific T-cell immunity, viral load, and clinical outcome in seropositive renal transplant recipients: a pilot study: CMV-specific immunity in renal transplant patients. Clin Transplant. (2009) 24:401–9. doi: 10.1111/j.1399-0012.2009.00976.x 19222507

[32] Yamada M Macedo C Louis K Shi T Landsittel D Nguyen C. Distinct association between chronic Epstein-Barr virus infection and T cell compartments from pediatric heart, kidney, and liver transplant recipients. Am J Transplant. (2023) 23(8):1145–58. doi: 10.1016/j.ajt.2023.05.007 37187296

[33] Egli A Binet I Binggeli S Jäger C Dumoulin A Schaub S. Cytomegalovirus-specific T-cell responses and viral replication in kidney transplant recipients. J Transl Med. (2008) 6:29. doi: 10.1186/1479-5876-6-29 18541023 PMC2432058

[34] Eid AJ Brown RA Arthurs SK Lahr BD Eckel-Passow JE Larson TS. A prospective longitudinal analysis of cytomegalovirus (CMV)-specific CD4+ and CD8+ T cells in kidney allograft recipients at risk of CMV infection: CMV-specific T-cell immunity after kidney transplant. Transplant Int. (2010) 23:506–13. doi: 10.1111/j.1432-2277.2009.01017.x 19951371

[35] Gerna G Lilleri D Fornara C Comolli G Lozza L Campana C. Monitoring of human cytomegalovirus-specific CD4+ and CD8+ T-cell immunity in patients receiving solid organ transplantation. Am J Transplant. (2006) 6:2356–64. doi: 10.1111/j.1600-6143.2006.01488.x 16889599

[36] Gerna G Lilleri D Chiesa A Zelini P Furione M Comolli G. Virologic and immunologic monitoring of cytomegalovirus to guide preemptive therapy in solid-organ transplantation. Am J Transplant. (2011) 11:2463–71. doi: 10.1111/j.1600-6143.2011.03636.x 21827612

[37] La Rosa C Limaye AP Krishnan A Longmate J Diamond DJ. Longitudinal assessment of cytomegalovirus (CMV)–specific immune responses in liver transplant recipients at high risk for late CMV disease. J Infect Dis. (2007) 195:633–44. doi: 10.1086/511307 17262704

[38] Radha R Jordan S Puliyanda D Bunnapradist S Petrosyan A Amet N. Cellular immune responses to cytomegalovirus in renal transplant recipients. Am J Transplant. (2005) 5:110–7. doi: 10.1111/j.1600-6143.2003.00647.x 15636618

[39] Rogers R Saharia K Chandorkar A Weiss ZF Vieira K Koo S. Clinical experience with a novel assay measuring cytomegalovirus (CMV)-specific CD4+ and CD8+ T-cell immunity by flow cytometry and intracellular cytokine staining to predict clinically significant CMV events. BMC Infect Dis. (2020) 20:58. doi: 10.1186/s12879-020-4787-4 31952516 PMC6969482

[40] Sester M Sester U Gärtner B Heine G Girndt M Mueller-Lantzsch N. Levels of virus-specific CD4 T cells correlate with cytomegalovirus control and predict virus-induced disease after renal transplantation. Transplant. (2001) 71:1287–94. doi: 10.1097/00007890-200105150-00018 11397964

[41] Sester U Gärtner BC Wilkens H Schwaab B Wössner R Kindermann I. Differences in CMV-specific T-cell levels and long-term susceptibility to CMV infection after kidney, heart and lung transplantation. Am J Transplant. (2005) 5:1483–9. doi: 10.1111/j.1600-6143.2005.00871.x 15888058

[42] Snyder LD Chan C Kwon D Yi JS Martissa JA Copeland CAF. Polyfunctional T-cell signatures to predict protection from cytomegalovirus after lung transplantation. Am J Respir Crit Care Med. (2016) 193:78–85. doi: 10.1164/rccm.201504-0733OC 26372850 PMC4731614

[43] Keoshkerian E Helbig K Beard M Zaunders J Seddiki N Kelleher A. A novel assay for detection of hepatitis C virus-specific effector CD4+ T cells via co-expression of CD25 and CD134. J Immunol Methods. (2012) 375:148–58. doi: 10.1016/j.jim.2011.10.004 22019644

[44] Sadler R Bateman EA Heath V Patel SY Schwingshackl PP Cullinane AC. Establishment of a healthy human range for the whole blood ‘OX40’ assay for the detection of antigen-specific CD4+ T cells by flow cytometry: Clinical validation of the ‘OX40’ assay for antigen-specifc CD4+ T cells. Cytometry. (2014) 86(5):350–61. doi: 10.1002/cytob.21165 24827553

[45] Arasaratnam RJ Tzannou I Gray T Aguayo-Hiraldo PI Kuvalekar M Naik S. Dynamics of virus-specific T cell immunity in pediatric liver transplant recipients. Am J Transplant. (2018) 18:2238–49. doi: 10.1111/ajt.14967 PMC6117219 29900673

[46] Imadome Ki Fukuda A Kawano F Imai Y Ichikawa S Mochizuki M. Effective control of Epstein-Barr virus infection following pediatric liver transplantation by monitoring of viral DNA load and lymphocyte surface markers. Pediatr Transplant. (2012) 16:748–57. doi: 10.1111/j.1399-3046.2012.01750.x 22764883

[47] Smets F Latinne D Bazin H Reding R Otte JB Buts JP. Ratio between Epstein-Barr viral load and anti-Epstein-Barr virus specific T-cell response as a predictive marker of posttransplant lymphoproliferative disease. Transplant. (2002) 73:1603–10. doi: 10.1097/00007890-200205270-00014 12042647

[48] Gotoh K Ito Y Ohta R Iwata S Nishiyama Y Nakamura T. Immunologic and virologic analyses in pediatric liver transplant recipients with chronic high Epstein-Barr virus loads. J Infect Dis. (2010) 202:461–9. doi: 10.1086/653737 20560768

[49] Ning RJ Xu XQ Chan KH Chiang AKS. Long-term carriers generate Epstein-Barr virus (EBV)-specific CD4(+) and CD8(+) polyfunctional T-cell responses which show immunodominance hierarchies of EBV proteins. Immunology. (2011) 134:161–71. doi: 10.1111/j.1365-2567.2011.03476.x PMC3194224 21896011

[50] Wilsdorf N Eiz-Vesper B Henke-Gendo C Diestelhorst J Oschlies I Hussein K. EBV-specific T-cell immunity in pediatric solid organ graft recipients with posttransplantation lymphoproliferative disease. Transplant. (2013) 95:247–55. doi: 10.1097/TP.0b013e318279968d 23222899

[51] Nagamata S Nagasaka M Kawabata A Kishimoto K Hasegawa D Kosaka Y. Human CD134 (OX40) expressed on T cells plays a key role for human herpesvirus 6B replication after allogeneic hematopoietic stem cell transplantation. J Clin Virol. (2018) 102:50–5. doi: 10.1016/j.jcv.2018.02.011 29494951

[52] Wang YL Fu YX Zhu ZJ Wang H Shen ZY. OX40 mRNA in peripheral blood as a biomarker of acute renal allograft rejection. Chin Med J (Engl). (2012) 125:3786–90. 23106874

[53] Díaz Fernández C Gámez Arance M de la Vega Bueno A Frauca Remacha E. Trasplante hepático pediátrico: indicaciones, técnicas quirúrgicas, complicaciones y tratamiento. Anales Pediatría. (2004) 60:42–55. doi: 10.1016/s1695-4033(04)78216-8 14718131

[54] Lovelace P Maecker HT. Multiparameter intracellular cytokine staining. In: Hawley TS Hawley RG, editors. Flow Cytometry Protocols, vol. 699 . Humana Press, Totowa, NJ (2011). p. 165–78. doi: 10.1007/978-1-61737-950-5_8 PMC4219546 21116983

[55] Welsh RM Selin LK. No one is naive: the significance of heterologous T-cell immunity. Nat Rev Immunol. (2002) 2:417–26. doi: 10.1038/nri820 12093008

[56] Rowntree LC Nguyen THO Halim H Purcell AW Rossjohn J Gras S. Inability to detect cross-reactive memory T cells challenges the frequency of heterologous immunity among common viruses. J Immunol. (2018) 200:3993–4003. doi: 10.4049/jimmunol.1800010 29735483

[57] Callan MF Tan L Annels N Ogg GS Wilson JD O’Callaghan CA. Direct visualization of antigen-specific CD8+ T cells during the primary immune response to Epstein-Barr virus In vivo. J Exp Med. (1998) 187:1395–402. doi: 10.1084/jem.187.9.1395 PMC2212279 9565632

[58] Lam JKP Hui KF Ning RJ Xu XQ Chan KH Chiang AKS. Emergence of CD4+ and CD8+ Polyfunctional T cell responses against immunodominant lytic and latent EBV antigens in children with primary EBV infection. Front Microbiol. (2018) 9:416. doi: 10.3389/fmicb.2018.00416 29599759 PMC5863510

[59] Lachmann R Bajwa M Vita S Smith H Cheek E Akbar A. Polyfunctional T cells accumulate in large human cytomegalovirus-specific T cell responses. J Virol. (2012) 86:1001–9. doi: 10.1128/JVI.00873-11 PMC3255847 22072753

[60] Soni MK Migliori E Fu J Assal A Chan HT Pan J. The prospect of universal coronavirus immunity: characterization of reciprocal and non-reciprocal T cell responses against SARS-CoV2 and common human coronaviruses. Front Immunol. (2023) 14:1212203. doi: 10.3389/fimmu.2023.1212203 37901229 PMC10612330

[61] Darrah PA Patel DT De Luca PM Lindsay RWB Davey DF Flynn BJ. Multifunctional TH1 cells define a correlate of vaccine-mediated protection against Leishmania major. Nat Med. (2007) 13:843–50. doi: 10.1038/nm1592 17558415

[62] Long HM Chagoury OL Leese AM Ryan GB James E Morton LT. MHC II tetramers visualize human CD4+ T cell responses to Epstein–Barr virus infection and demonstrate atypical kinetics of the nuclear antigen EBNA1 response. J Exp Med. (2013) 210:933–49. doi: 10.1084/jem.20121437 PMC3646497 23569328

[63] Gasper DJ Tejera MM Suresh M. CD4 T-cell memory generation and maintenance. Crit Rev Immunol. (2014) 34:121–46. doi: 10.1615/CritRevImmunol.2014010373 PMC4062920 24940912

[64] Mahnke YD Brodie TM Sallusto F Roederer M Lugli E. The who’s who of T -cell differentiation: Human memory T -cell subsets. Eur J Immunol. (2013) 43:2797–809. doi: 10.1002/eji.201343751 24258910

[65] Wang Y Qiu F Xu Y Hou X Zhang Z Huang L. Stem cell-like memory T cells: The generation and application. J Leukocyte Biol. (2021) 110:1209–23. doi: 10.1002/JLB.5MR0321-145R 34402104

[66] Palianina D Mietz J Stühler C Arnold B Bantug G Münz C. Stem cell memory EBV-specific T cells control post-transplant lymphoproliferative disease and persist in *vivo* . Immunology. (2023) 10(34):eado2048. doi: 10.1101/2023.05.30.542809 PMC11343021 39178248

[67] Amyes E Hatton C Montamat-Sicotte D Gudgeon N Rickinson AB McMichael AJ. Characterization of the CD4+ T cell response to epstein-barr virus during primary and persistent infection. J Exp Med. (2003) 198:903–11. doi: 10.1084/jem.20022058 PMC2194204 12975456

[68] Liu L Cui J You Y Shi W Zou P Chen L. Expression of CD134 on CD4+ T cells reflects the immunosuppressive state after allo-HCT by revealing the intensity of T cell activation. Int J Clin Exp Med. (2016) 9:17782–91.

[69] Lamb LS Abhyankar SA Hazlett L O’Neal W Folk RS Vogt S. Expression of CD134 (0X-40) on T cells during the first 100 days following allogeneic bone marrow transplantation as a marker for lymphocyte activation and therapy-resistant graft-versus-host disease. Cytometry. (1999) 38:238–43. doi: 10.1002/(SICI)1097-0320(19991015)38:5<238::AID-CYTO6>3.0.CO;2-O 10516610

