# Supplementary figures and images for: Epstein-Barr virus-specific T-cell response in pediatric liver transplant recipients: a cross-sectional study by multiparametric flow cytometry
Source: Front Immunol. 2024 Oct 24;15:1479472. doi: 10.3389/fimmu.2024.1479472 (PMC11540634; doi:10.3389/fimmu.2024.1479472)

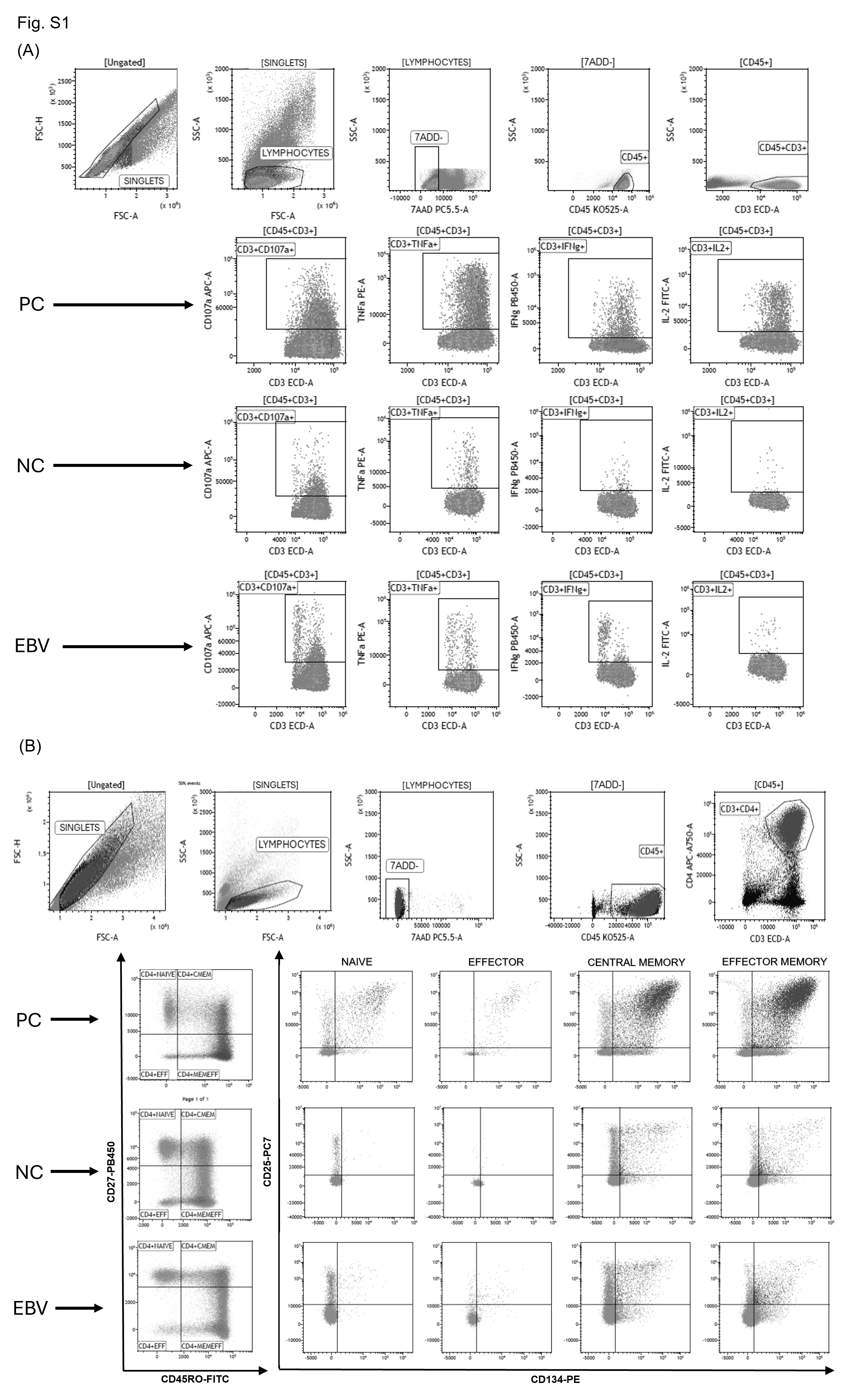

Supplement: Supplementary Figure 1 — Flow-cytometry gating strategy for (A) intracellular cytokine staining or (B) activation-induced marker staining in a representative healthy control. Cells were left unstimulated (NC) or stimulated using a positive control (PC) or Epstein-Barr virus peptides (EBV). [file Image1.tif]
