# Supplementary material for: Epstein-Barr virus-specific T-cell response in pediatric liver transplant recipients: a cross-sectional study by multiparametric flow cytometry
Source: Front Immunol. 2024 Oct 24;15:1479472. doi: 10.3389/fimmu.2024.1479472 (PMC11540634; doi:10.3389/fimmu.2024.1479472)
Supplement: Supplementary file 2 [file Table1.docx]

**Supplementary Methods**

*2.2. Intracellular cytokine staining*

In brief, 1x10^6^ PBMCs in TexMACS™ medium 10% fetal bovine serum (FBS) were stimulated with 0.1µg EBV peptides (PepTivator® EBV, Miltenyi, Germany) for 6 hours at 37°C. As a negative control, PBMCs were incubated without stimulation. Dynabeads® Human T-Activator CD3/CD28 (Life Technologies, Norway) was used as positive control stimulus. Brefeldin A (Sigma, USA) and monoclonal antibody CD107a-APC (Beckman Coulter) were added after 2 hours of stimulation. Four hours later, cells were washed with CliniMACS® PBS/EDTA Buffer and stained with anti-CD45-KO, anti-CD3-ECD, anti-CD4-APC750 and anti-CD8-APC700 monoclonal antibodies (Beckman Coulter, USA). Live cells were marked with 7-aminoactinomycin D (7-AAD, Beckman Coulter, USA). Following PBMCs permeabilization (Beckman Coulter, USA), cells were intracellularly stained with anti-IFNɣ-PB, anti-interleukin-2-FITC (IL2) and anti-TNFα-PE monoclonal antibodies (Beckman Coulter, USA). Cell staining was analyzed by flow cytometry on DxFLEX and the resulting data were analyzed by Kaluza software (Beckman Coulter, USA).

Flow cytometry assays were considered as evaluable when average acquisition of live lymphocytes was higher than 1x10^4^ and positive control stimulus was over 0.10% CD3+IFNɣ+ cells.

*2.3. Activation-induced cell marker staining*

Briefly, 1x10^6^ PBMCs in TexMACS™ medium 10% FBS were stimulated with 0.1µg EBV peptides (PepTivator® EBV, Miltenyi, Germany) for 48 hours at 37°C. As a negative control, PBMCs were incubated without stimulation. Dynabeads® Human T-Activator CD3/CD28 (Life Technologies, Norway) was used as positive control stimulus. After incubation, cells were washed with CliniMACS® PBS/EDTA Buffer and stained with anti-CD45-KO, anti-CD3-ECD, anti-CD4-APC750, anti-CD45RO-FITC, anti-CD25-PC7, anti-CD27-PB (Beckman Coulter, USA) and anti-CD134-PE (Cytognos, Spain) monoclonal antibodies. Live cells were marked with 7-AAD (Beckman Coulter, USA). Cell staining was analyzed by flow cytometry on DxFLEX and the resulting data were analyzed by Kaluza software (Beckman Coulter, USA).

Flow cytometry assays were considered as evaluable when average acquisition of live lymphocytes was higher than 1x10^4^ and positive control stimulus was over 7.50% CD4+CD25+CD134+ cells.

*2.4 Immunophenotype analysis*

Briefly, 5x10^5^ PBMCs resuspended in TexMACS™ medium 10% FBS were stained with anti-CD45-KO, anti-CD3-ECD, anti-CD4-APC750, anti-CD8-APC700, anti-CD19-PC7, anti-CD16-APC and anti-CD56-APC monoclonal antibodies (Beckman Coulter, USA). Cell staining was analyzed by flow cytometry on DxFLEX and the resulting data were analyzed by Kaluza software (Beckman Coulter, USA).

**Table S1. Median of the integrated median fluorescence intensity (iMFI) for each marker (CD107a, IFNɣ, IL2 or TNFα) in both CD4+ and CD8+ T cells. Data is reported for monofunctional (mono) and polyfunctional (poly) response in EBV-seropositive adult healthy controls (HC) and immunosuppressed paediatric liver-transplanted patients, categorized as positive/negative serology status (IP-S^POS^ and IP-S^NEG^, respectively).**

| **iMFI** | | **HC (n=24)** | | **P-value** | **IP-S^NEG^ (n=6)** | | **P-value** | **IP-S^POS^ (n=32)** | | **P-value** |
| --- | --- | --- | --- | --- | --- | --- | --- | --- | --- | --- |
| **T-cell subset** | **Parameter** | **Mono** | **Poly** |  | **Mono** | **Poly** |  | **Mono** | **Poly** |  |
| **CD4+** | CD107a+ | 1,434 | 1,060 | 0.89 | 0 | 2,588 | 0.27 | 0 | 1,813 | 0.08 |
|  | IFNɣ+ | 115 | 475 | **<0.001** | 38 | 49 | 0.67 | 31 | 179 | **<0.001** |
|  | IL2+ | 0 | 122 | **<0.001** | 31 | 237 | 0.52 | 0 | 93 | **0.005** |
|  | TNFα+ | 118 | 1,248 | **0.03** | 60 | 1,838 | **0.04** | 208 | 1,422 | **0.002** |
| **CD8+** | CD107a+ | 0 | 17,538 | **<0.001** | 0 | 373 | 0.11 | 3,812 | 42,771 | **0.002** |
|  | IFNɣ+ | 280 | 3,366 | **<0.001** | 160 | 36 | 0.27 | 362 | 7,714 | **<0.001** |
|  | IL2+ | 30 | 377 | **0.005** | 51 | 0 | 0.15 | 20 | 424 | **<0.001** |
|  | TNFα+ | 288 | 7,370 | **<0.001** | 122 | 0 | 0.42 | 181 | 8,307 | **<0.001** |
